# Biliary Microbiome Dysbiosis Is Associated with Susceptibility to Post-ERCP Pancreatitis

**DOI:** 10.1055/a-2949-6689

**Published:** 2026-09-10

**Authors:** Jonathan Y. Xia, Srinadh Komanduri, Rajesh N. Keswani, Terrance R. Rodrigues, Jasmine Sinha, Arvind Rengarajan, Benjamin M. Moy, Sarah F. Gruber, You-Jia Chen, Chelsea Hepler, Arthur Prindle, A. Aziz Aadam

**Affiliations:** 1 Division of Gastroenterology and Hepatology 1259 University of MichiganAnn ArborUnited States; 2 Division of Gastroenterology and Hepatology 12244 Northwestern University Feinberg School of MedicineChicagoIllinoisUnited States; 3 Division of Gastroenterology and Hepatology 12275 Washington University in St Louis School of MedicineSt. LouisMissouriUnited States; 4 Department of Molecular Integrative Physiology 1259 University of MichiganAnn ArborUnited States; 5 Department of Biochemistry and Molecular Genetics and Center for Synthetic Biology 3270 Northwestern UniversityEvanstonUnited States

**Keywords:** pancreatobiliary (ERCP/PTCD), ERC topics, quality and logistical aspects, performance and complications

## Abstract

**Background & Aims** Pancreatitis remains the most common adverse event following endoscopic retrograde cholangiopancreatography (ERCP), yet current risk stratification relies primarily on clinical and procedural factors. The present study aims to examine bile-based microbiome signatures as a potential link between nonprocedure patient-level risk factor for pancreatitis susceptibility after ERCP.

**Methods** We performed analysis for rates of post-ERCP pancreatitis of a prospective single center cohort of patients undergoing ERCP ( *n* = 148) with bile collected at the time of the procedure. Microbial profiling was performed using 16S rRNA sequencing. Alpha diversity, beta diversity, differential abundance analyses, and metabolic pathway analysis were evaluated between patients with and without post-ERCP pancreatitis (PEP).

**Results** Patients with PEP demonstrated significantly reduced biliary microbial diversity and distinct composition compared with those without pancreatitis. Differential abundance analysis identified multiple depleted genera in the PEP group. Functional profiling revealed broad reductions in microbial metabolic pathways associated with PEP predominantly involving biosynthetic and central metabolic functions. ROC curve analysis demonstrated that differentially abundant bile microbes can discriminate patients with PEP from those without pancreatitis.

**Discussion** Patients who develop PEP harbor a distinct and functionally depleted biliary microbiome. These findings parallel prior observations of gut dysbiosis in acute pancreatitis and suggest that the biliary microbiome may represent a biologic pathway associated with PEP susceptibility.

## Introduction

Post-endoscopic retrograde cholangiopancreatography pancreatitis (PEP) remains the most common adverse event following endoscopic retrograde cholangiopancreatography (ERCP), occurring in approximately 3–10% patients on average and up to 15–20% in high-risk populations. Despite advances in procedural techniques and prevention strategies, PEP continues to be a major problem in clinical practice resulting in prolonged hospitalization and increased health care utilization. Furthermore, recent studies have shown a rising incidence rate of PEP and associated mortality in the United States. 1 2

Recent studies have identified nonprocedural patient-level risk factors, such as female sex and younger age, that are associated with PEP. 3 Despite the consistency of these clinical associations, these patient-level variables do not fully explain why some patients appear intrinsically more susceptible to pancreatic injury after ERCP. This gap suggests that there are biologic factors beyond standard demographic and clinical variables that may contribute to inter-individual vulnerability to PEP.

A growing body of evidence supports a close relationship between alterations of the gut microbiome and acute pancreatitis. Patients with acute pancreatitis exhibit gut dysbiosis characterized by reduced microbial diversity and shifts in taxanomic composition that have been shown to correlate with pancreatitis severity. 4 Furthermore, microcirculatory injury and hypovolemia seen in acute pancreatitis can lead to intestinal mucosal ischemia and disruption of the intestinal barrier resulting in gut bacterial translocation of the pancreas. 5 This results in amplification of local and systemic inflammatory responses in acute pancreatitis. 5 6 Prior work showed substantial overlap between the small bowel and bile duct microbiomes, suggesting that biliary microbes may influence inflammatory susceptibility of the pancreas. 7 8 Given the anatomic continuity of the biliary tract with the pancreas and its direct perturbation during ERCP, we hypothesized that biliary microbial composition and function may represent one biologic pathway linking patient-level risk factors to PEP.

## Results

A total of 148 patients were included in our cohort of patients undergoing ERCP. All patients underwent ERCP for indications including elevated liver enzymes, abnormal imaging, or biliary obstruction. Among 148 patients, 11 developed post-ERCP pancreatitis, defined as lipase elevation ≥3× the upper limit of normal, abdominal pain, or imaging evidence of pancreatitis. Demographics including median age, preprocedural antibiotics administration, presence of cholangitis, native papilla status, and total bilirubin pre-ERCP were overall comparable between patients with and without post-ERCP pancreatitis ( Table 1 ). In contrast, several procedural factors differed between the PEP and no pancreatitis group. Patients with PEP were more likely to have contrast injection into or wire passage into the pancreatic duct, undergo double-wire technique, and receive pancreatic duct stent placement ( Table 2 ). Needle-knife use and rectal indomethacin administration among patients with native papilla did not differ significantly between groups, whereas fellow participation was less common in cases complicated by PEP ( Table 2 ).

**Table 1:** Cohort demographics in patients with and without post-ERCP pancreatitis.

	PEP ( *n* = 11)	No Pancreatitis ( *n* = 137)
Age (median with range)	67 (IQR 50–71.5)	59 (IQR 44–68)
% Female	27.3 ( *n* = 3)	54.7 ( *n* = 75)
Total bilirubin before ERCP (mg/dL)	1.9 (IQR 1.2–3.45)	2.0 (IQR 0.8–4.95)
% Native papilla	81.8 ( *n* = 9)	75.2 ( *n* = 103)
% Pre-ERCP antibiotics	36.3 ( *n* = 4)	45.9 ( *n* = 63)
% Cholangitis	9.1 ( *n* = 1)	11.7 ( *n* = 16)

**Table 2:** Procedural characteristics in patients with and without post-ERCP pancreatitis.

	PEP ( *n* = 11)	No Pancreatitis ( *n* = 137)
% Fellow in case	36.4 (4/11)	75.9 (104/137)
% Contrast injected into PD or wire into PD	63.6 (7/11)	18.9 (26/137)*
% Double wire technique	45.5 (5/11)	8.8 (12/137)*
% Rectal indomethacin (native papilla only)	30.0 (3/10)	19.2 (20/104)
% PD stent placement	27.3 (3/11)	6.6 (9/137)*
% Needle knife used	0 (0/11)	2.9 (4/137)

* *p* < 0.05 in Fisher’s exact test.

Of the 11 patients with PEP, only 1 patient had a documented history of acute pancreatitis ( **Supplementary Table 1** ). Furthermore, eight patients required hospital admission for management of PEP, with lengths of stay ranging from 1 to 45 days, while one patient who was already hospitalized had their admission prolonged by 2 days because of pancreatitis ( **Supplementary Table 1** ).

Previous studies have demonstrated evidence of gut dysbiosis in patients with acute pancreatitis compared to healthy controls. 5 We identified the five phyla of *Proteobacteria, Firmicutes, Actinobacteriota, Fusobacteriota* , and *Bacteriodota* accounted for the majority of the microbiome in bile of patients with and without PEP ( Fig. 1A ). A total of 502 genera were identified, the dominant genera seen in bile from all groups were *Escherichia, Shigella* , *Streptococcus* , *Prevotella* , *Enterobacteriaceae* , and *Enterococcus* ( Fig. 1B ). *Escherichia* and *Shigella* was the most abundant genera seen in bile of both groups comprising of 61.4% of the bile microbiome in PEP and 29.6% of no pancreatitis ( Fig. 1B ). Furthermore, *Enterococcus* was more abundant in bile of PEP (11.9%) compared to no pancreatitis (3.2%), while *Enterobacteriaceae* was enriched in patients with no pancreatitis (13.2%) compared to PEP (3.2%).

**Fig. 1:**
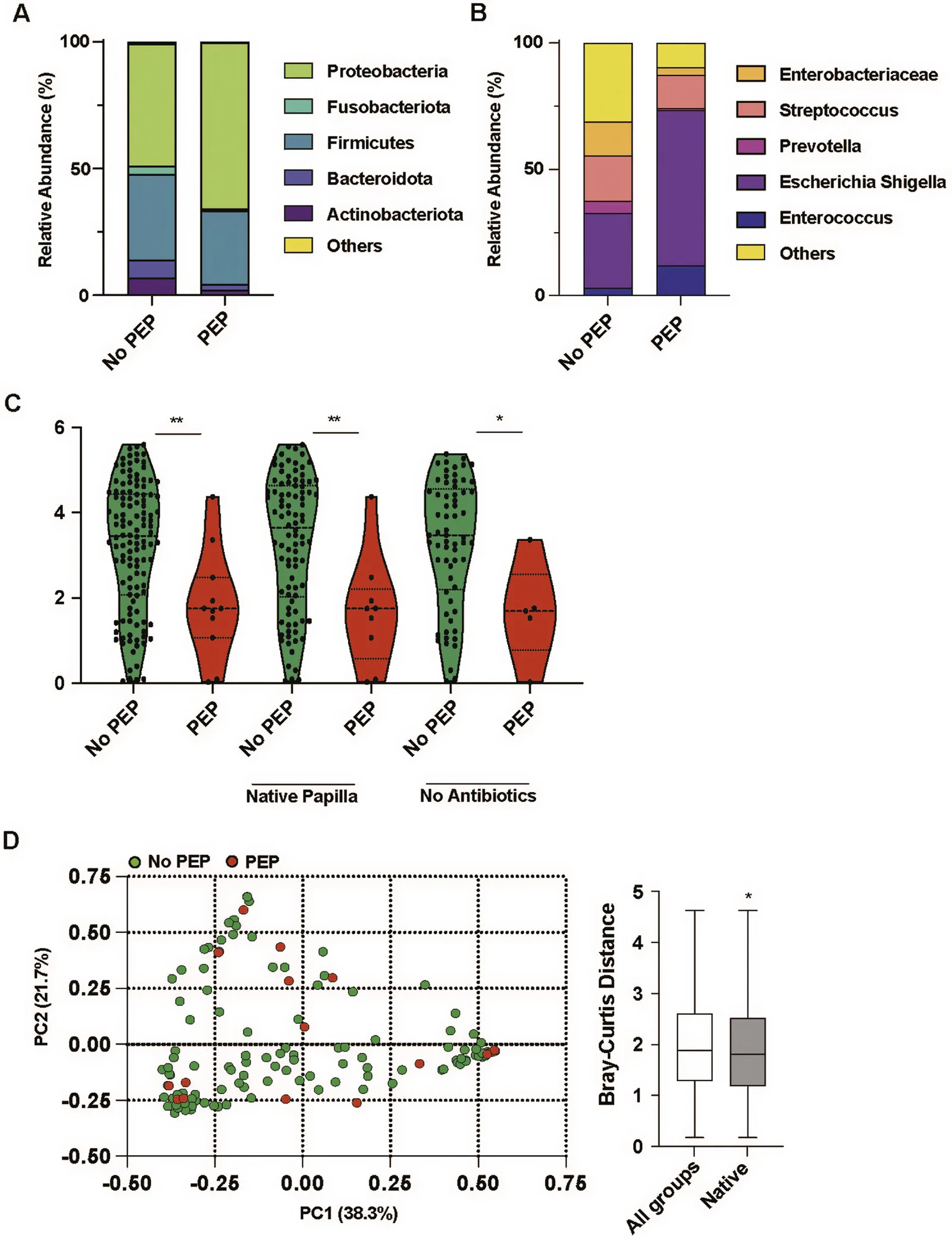
Bile microbiome composition is distinct in patients with post-ERCP pancreatitis (PEP) compared to patients without pancreatitis. ( **A** ) Relative abundance distributions of the top five phyla in the bile samples from patients with PEP and no pancreatitis. ( **B** ) Relative abundance distributions of the top five genus in the bile samples from patients with PEP and no pancreatitis. ( **C** ) Alpha-diversity calculated using Shannon diversity index comparing patients with PEP and no pancreatitis. ( **D** ) Principal coordinate analysis plot and Beta-diversity calculated using Bray-Curtis distance. * *p* < 0.05, ** *p* < 0.01 for PERMANOVA for indicated comparisons.

To assess alpha-diversity, a measure of the distribution of microbes within a sample, we utilized Shannon entropy index, a metric that incorporates both the number of taxa present and the evenness of their distribution in the sample. Alpha-diversity was significantly lower in the PEP group ( Fig. 1C ), indicating overall reduced microbial diversity. Sub-group analysis showed that these differences in diversity persisted after accounting for exposure to pre-ERCP antibiotics and patient’s native papilla status ( Fig. 1C ). The overall microbiota composition (β-diversity) of bile from patients with PEP did not differ compared to those without PEP ( Fig. 1D ) as assessed by PERMANOVA of Bray-Curtis distance. However, after accounting for native papilla status, we observed significant differences in composition in the microbiome of bile from patients of PEP compared to patients without pancreatitis ( Fig. 1D ). Collectively, these results indicate that patients who develop PEP exhibit dysbiosis of the biliary microbial ecosystem characterized by reduced diversity and altered community structure.

To identify microbial markers of PEP, we next performed differential abundance analysis of our 16S rRNA dataset on the level of genus. Multivariable differential abundance analysis utilizing MaAsLin2 identified the genera *Gemella* , *Neisseria* , and *Prevotella* to be differentially abundant in bile of patients of PPE (FDR < 0.01, Fig. 2A , **Supplementary Fig. 1A** ). Considering that native papilla status is highly relevant in the context of post-ERCP pancreatitis, we next performed differential abundance analysis after accounting for native papilla status in our patient cohort. 3 We found the genera *Alloprevotella, Fusobacterium* , *Granulicatella, Veillonella* , and *Actinomyces* to be differentially abundant in bile of patients of PPE in addition to the initial 3 genera identified on MaAsLin2 analysis ( Fig. 2A , **Supplementary Fig. 1A** ). Furthermore, all differentially abundant genera were depleted in the bile of patients of PEP.

**Fig. 2:**
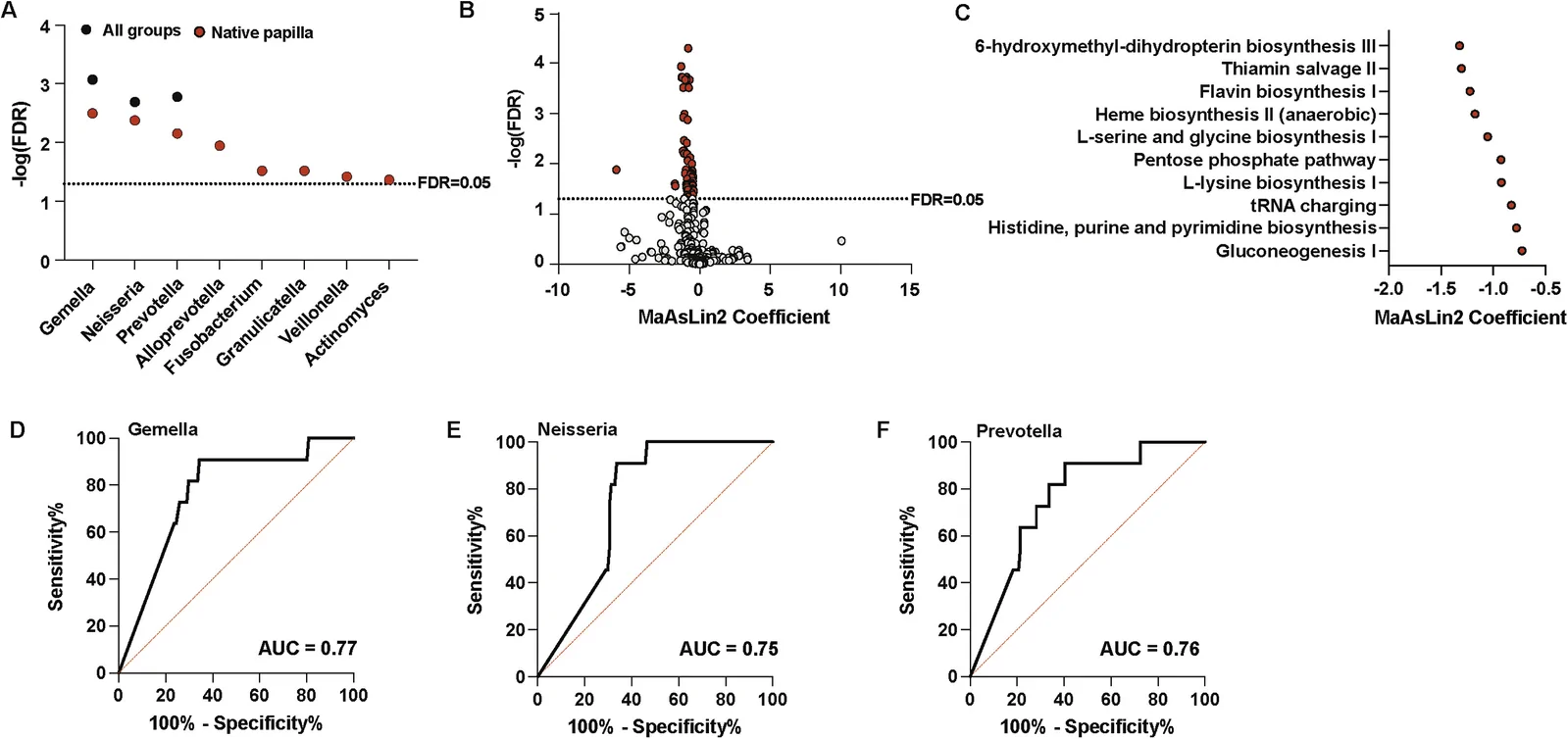
Functional profiling of bile microbiome in patients with PEP shows depletion of pathways critical for maintaining microbial homeostasis. ( **A** ) Differential abundant genera comparing PEP and no pancreatitis identified through MaAsLin2 of all patients (black) and those with native papilla (red). ( **B** ) Volcano plot of all statistically significant (FDR < 0.05) bacterial pathways in patients with PEP identified through MaAsLin2. ( **C** ) Forrest plot of the 10 most significant bacterial pathways associated with PEP. ( **D** ) ROC curve for discrimination of PEP versus no pancreatitis using bile relative abundance of *Gemella* in bile ( *p* = 0.0029, 95% CI 0.64–0.90). ( **E** ) ROC curve for discrimination of PEP versus no pancreatitis using relative abundance of *Neisseria* in bile ( *p* = 0.0058, 95% CI 0.66–0.84). ( **F** ) ROC curve for discrimination of PEP versus no pancreatitis using relative abundance of *Prevotella* in bile ( *p* = 0.0041, 95% CI 0.64–0.88).

To explore potential functional links between biliary microbial changes and PEP, we performed functional profiling using PICRUSt2 followed by multivariable association testing with MaAsLin2. After false discovery rate correction (FDR < 0.05), 73 bacterial metabolic pathways demonstrated statistically significant association with the pancreatitis group ( Fig. 2B ). A profound global shift in the functional landscape was observed, dominated almost exclusively by a loss of microbial metabolic capacity in the PEP group. Of the significant features, the vast majority exhibited negative coefficients, indicating a systematic depletion of core commensal functions. Notably, forest plot demonstrated the most significant associations involving pathways related to core bacterial growth and biosynthetic metabolism functions, including tRNA charging, amino acid biosynthesis, vitamin and cofactor metabolism, and central carbon metabolism ( Fig. 2C ). Categorization of significant pathways by metabolic sector revealed that biosynthesis was the most heavily impacted functional block, followed by energy/central metabolism, degradation/utilization, and salvage pathways ( **Supplemental Fig. 1B** ). The widespread reduction in these pathways suggests a "functional collapse" of the gut microbiota in patients with PEP, where pathways critical for maintaining microbial homeostasis are severely diminished.

To explore the potential clinical relevance of differentially abundant bile microbes, we next evaluated their ability to discriminate patients with PEP from those without pancreatitis. Receiver operating characteristic (ROC) curve analysis in the full cohort of 148 patients demonstrated that all eight previously identified differentially abundant biliary microbes can significantly ( *p* < 0.05) discriminate patients with PEP from those without pancreatitis. Specifically, we found *Gemella* , *Neisseria* , and *Prevotella* demonstrated the strongest performance with AUCs of 0.77, 0.75, and 0.76, respectively ( Fig. 2D–F , **Supplementary Fig. 1C** ).

## Discussion

Our data demonstrate a distinct and dysbiotic biliary microbiome in patients with PEP compared to those without pancreatitis after ERCP. Overall, the bile microbiome in patients with PEP had reduced diversity and altered community composition with functional profiling demonstrating loss of microbial metabolic capacity important for maintaining community homeostasis. Selected differentially abundant microbes also showed discriminatory performance for PEP on ROC analysis, supporting the potential utility of bile microbial features as candidate biomarkers for risk stratification. Our findings raise the possibility that the biliary microbiome is an additional nonprocedural factor associated with PEP susceptibility that could complement established clinical and procedural variables in future risk-stratification models.

Patients with acute pancreatitis have been shown to exhibit gut dysbiosis and intestinal barrier dysfunction, which have been implicated as amplifiers of pancreatic inflammation in both murine and human studies. 9 10 11 Prior studies have also demonstrated that the bile duct harbors a distinct microbiome associated with pancreaticobiliary disorders such as primary sclerosing cholangitis, cholangiocarcinoma, and pancreas cancer. 7 8 12 13 Our findings are consistent with these findings, demonstrating that patients who develop PEP similarly exhibit reduced microbial diversity and broad depletion of commensal-associated functions within bile. Similar to previous studies, native papilla status appears to play an important role in determining biliary microbiome compositional differences between patients with PEP and those without pancreatitis. 8 Furthermore, functional profiling of the bile microbiome exhibited global reductions in pathways related to bacterial biosynthesis and metabolism in patients with PEP suggestive of reduced microbial functional capacity. This pattern parallels emerging concepts in acute pancreatitis suggesting that loss of microbial diversity and metabolic homeostatic capacity, rather than simple pathogen expansion, may be an important nonprocedural feature contributing to heightened inflammatory vulnerability of the pancreas. 4 10

Although there are many studies demonstrating the association of microbiome dysbiosis with acute pancreatitis, there is still a lack of studies on its role specifically in PEP. All significant differentially abundant biliary microbes were able to significantly discriminate patients with PEP from those without pancreatitis on ROC curve analysis with *Gemella* , *Neisseria* , and *Prevotella* having the strongest performance (AUC > 0.75). From a clinical standpoint, our data raise the possibility that bile-based microbial features could serve as candidate biomarkers for PEP risk stratification. Importantly, the observed associations persisted after accounting for key clinical covariates, including native papilla status and pre-ERCP antibiotic exposure. Current risk stratification relies primarily on clinical and procedural variables, which incompletely account for individual susceptibility to inflammatory injury. As bile can be readily obtained at the time of ERCP, point-of-care microbial profiling could augment existing risk models or help identify patients who may benefit from intensified prophylactic strategies.

Several limitations for this study should be considered. This was a single-center study with a modest but clinically accurate number of PEP events (7.4%), which may limit generalizability and statistical power. In addition, the study population was heterogeneous and included patients with prior sphincterotomy and preprocedural antibiotic exposure, both of which could alter the biliary microbiome. Also, functional inference was performed using PICRUSt2 rather than metagenomic sequencing and therefore reflects predicted rather than directly measured metabolic activity. Finally, although we adjusted for key confounders, residual confounding related to procedural complexity and unmeasured host factors remains possible.

Susceptibility to PEP likely reflects an interaction between underlying patient-level demographics and procedural-related factors. In our cohort, pancreatic duct instrumentation and double-wire cannulation were more frequent among patients who developed PEP, whereas measured demographic characteristics were similar between groups. However, these factors do not fully explain why pancreatitis develops in only a subset of patients exposed to similar procedural-related stress. As microbial composition varies with age and sex, microbiome-associated differences may represent one biologic nonprocedural mechanism contributing to demographic variation in PEP risk. Differentially abundant microbes also showed potential translational utility as candidate markers of PEP susceptibility. Given the anatomic proximity of bile to the pancreas and the routine accessibility of bile during ERCP, further mechanistic and validation studies are needed to determine whether bile microbial signatures can clarify the biologic basis of patient-level susceptibility to PEP and improve risk-stratification.

## Materials and Methods

### Patient Population and Sample Collection

In this prospective single center study, bile was obtained from inpatients and outpatients undergoing ERCP from 2021 to 2024 at Northwestern Memorial Hospital for indication of biliary obstruction due to malignant biliary stricture, benign biliary diseases (i.e., choledocholithiasis, sphincter of Oddi dysfunction), primary sclerosing cholangitis, and transplant related strictures. A single bile sample from a single procedure encounter was collected in all patients that were enrolled in the study (148 total). No samples were collected from patients with altered anatomy. The present study was approved under the Institutional Review Board-approved studies STU00203172 and STU215612.

After consent was obtained prior to procedure, bile sample was collected and stored as previously described. 8 In brief, to prevent contamination from duodenal fluid, a 3–5 mL bile sample was aspirated after deep cannulation of the bile duct during ERCP before contrast injection with a sterile sphincterotome or balloon catheter. Antibiotics required for outpatient ERCP for patients with a history of transplant or primary sclerosing cholangitis were applied intravenously after bile samples were obtained, if they were indicated for the procedure. Bile samples were then aliquoted to cryovials and snap-frozen in liquid nitrogen. Samples were then stored in an −80 °C freezer until processing for 16S sequencing.

### DNA Preparation and 16S Sequencing

DNA extraction from bile and the 16S sequencing methods and analysis were performed by the Genomics and Microbiome Core Facility (GMCF) at Rush University as previously described. 8 Details on the sequencing methods and statistical analysis are provided in the Supplementary material.

### Statistical Analysis

Patient cohort and procedural-related data analyses were done using Fisher’s exact test for binary variables and the Mann–Whitney test for continuous variables with Graphpad Prism. The 16S sequencing data analysis was performed by the Rush Microbiome Core as previously described. 8 In brief, to account for differences in sequence depth after quality filtering and decontamination, samples were rarefied to a depth of 9,000 reads prior to analysis. Alpha-diversity metrics (Shannon Index) was calculated using q2-diversity. To assess the microbial community composition, we conducted a Permutational Multivariate Analysis of Variance (PERMANOVA) within QIIME2 environment with 9999 permutations on feature levels using Aitchison distance, a linear measure of sample dissimilarity for compositional data. Differentially abundant phylum and genus level taxa between pairwise groups were identified using MaAsLin2. 14 Functional metabolic profiles of bacterial communities were analyzed using PICRUSt2, and analysis was performed using MaAsLin2. Individual microbial biomarkers were assessed with receiver operating characteristic (ROC) curves using GraphPad Prism. Further details on statistical analysis are provided in the Supplementary Methods.

## Data Availability

Any additional information required to re-analyze the data reported in this paper is available from the corresponding author upon request.

## Supplementary Material

Ergänzendes Material Supplementary Material
