# Supplementary material for: Biliary Microbiome Dysbiosis Is Associated with Susceptibility to Post-ERCP Pancreatitis
Source: Endosc Int Open. 2026 Sep 10;14:a29496689. doi: 10.1055/a-2949-6689 (PMC13641221; doi:10.1055/a-2949-6689)
Supplement: Supplementary file 1 [file 10-1055-a-2949-6689_29499875.pdf]

Supplemental Materials

Title: Biliary Microbiome Dysbiosis Is Associated with Susceptibility to Post-ERCP Pancreatitis

Authors: Jonathan Y. Xia[1*], Srinadh Komanduri[2], Rajesh N. Keswani[2], Terrance R. Rodrigues[2], Jasmine Sinha[2], Arvind Rengarajan[3], Benjamin M. Moy[2], Sarah F. Gruber[2], You-jia Chen[4], Chelsea Hepler[4], Arthur Prindle[5], A. Aziz Aadam[2]

Affiliations:
[1] Division of Gastroenterology & Hepatology, Department of Medicine, University of Michigan, Ann Arbor, MI, USA.
[2] Division of Gastroenterology & Hepatology, Department of Medicine, Feinberg School of Medicine, Northwestern University, Chicago, IL, USA.
[3] Division of Gastroenterology & Hepatology, Department of Medicine, Washington University School of Medicine, St. Louis, MO, USA.
[4] Department of Molecular Integrative Physiology, University of Michigan, Ann Arbor, MI, USA.
[5] Department of Biochemistry and Molecular Genetics and Center for Synthetic Biology, Northwestern University, Evanston, IL, USA.

***Correspondence:** Jonathan Xia, 1(708)-616-6020, jyxia@med.umich.edu, 1500 E Medical Center Dr Floor 3 Reception D, Ann Arbor MI, 48109

Supplemental Figure 1

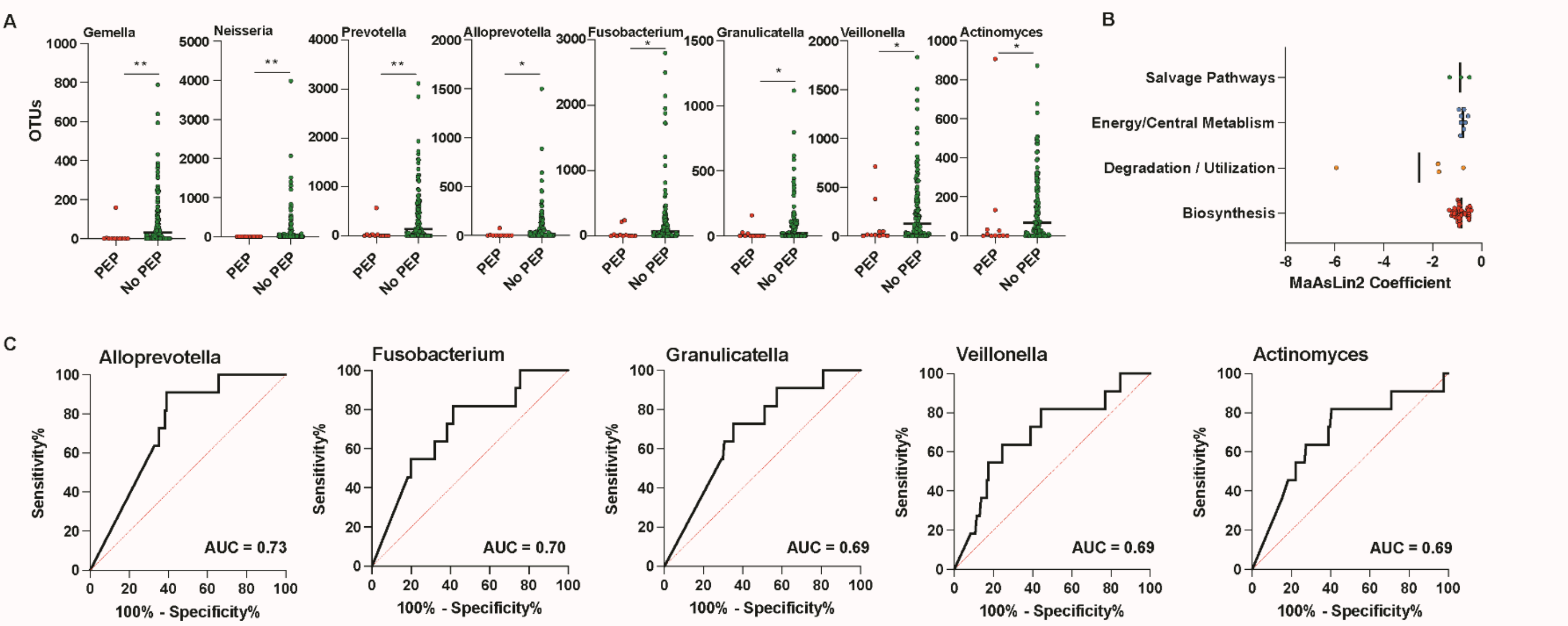

Supplemental Figure 1. Differential abundance and pathway analysis of bile fluid collected during ERCP of patients with and without post-ERCP pancreatitis.
(A) Differentially abundant genera (FDR<0.05) determined by MaAsLin2 comparing PEP and no pancreatitis pantients (B) Categorization of significant pathways determined by MaAsLin2 (FDR <0.05) by functional blocks of biosynthesis (n=58), degredation/utilization (n=4), energy/central metabolism (n=8), salvage pathways (n=3). (C) ROC curve for discriminating pateints with PEP and no PEP using the relative abundance of the genus *Alloprevotella (p = 0.01, CI 0.62-0.85), Fusobacterium (p=0.025, CI 0.551-0.85), Granulicatella (p=0.037, CI 0.55-0.83), Veillonella (p=0.033, CI 0.53-0.86), and Actinomyces (p=0.039, CI 0.51-0.86).*

Supplementary Table 1: Characteristics of patients with PEP

PEP patient	Necrotizing pancreatitis on Imaging	If outpatient was patient re-admitted? Days of admission	If inpatient, was admission prolonged due to this	History of pancreatitis
1	yes	NA	NA	yes
2	no	yes, 5	NA	no
3	no	yes, 2	NA	no
4	no	yes, 45	NA	no
5	no	yes, 3	NA	no
6	no	yes, 1	NA	no
7	No Imaging done	NA	NA	no
8	No Imaging done	yes, 4	NA	no

Supplementary Methods

DNA Preparation and 16S Sequencing

Microbial DNA was extracted from 250 µL of human bile using a modified QIAGEN DNeasy PowerSoil Pro kits where the DNA binding spin column steps were replaced with DNA purification through Ampure magnetic beads (Beckman Coulter). In brief, after cell lysis step, the lysates 1:1 ratio of magnetic beads were mixed with the lysate. This mixture was then placed on the magnetic stand and washed twice with freshly made 80% ethanol. Residual ethanol was removed, and beads were air-dried on 2 minutes. The beads were removed from the magnetic stand and resuspended in 10 uL of sterile water. The was then placed back on the magnetic stands and supernatant was placed in new Eppendorf tube. The DNA extraction protocol was confirmed using human stool samples as positive controls and sterile water as negative controls.

16S Sequencing

Genomic DNA was PCR amplified with primers CS1_515F and CS2_806R (modified from the primer set employed by the Earth Microbiome Project (EMP; ACACTGACGACATGGTTCTACAGTGTGYCAGCMGCCGCGGTAA and TACGGTAGCAGAGACTTGGTCTGGACTACNVGGGTWTCTAAT, respectively – underlined regions represent linker sequences) targeting the V4 region of microbial small subunit ribosomal RNA genes. Amplicons were generated using a two-stage PCR amplification protocol like that described previously [1]. PCR products were pooled in equal volumes then purified using a Ampure beads (Beckman Coulter). Bead-purified pool of DNA fragments was further purified with a Pippin

Prep device (Sage Science) with 2% agarose gel. Size selected DNA fragments (360-440 bp range; target size 400 bp) from the PippinPrep were sequenced with a 10% phiX spike-in on an Illumina Miniseq sequencer employing a mid-output flow cell (2x154 paired-end reads). Based on the results of the MiniSeq sequencing run, samples were re-pooled to balance output evenly among samples. The pooled products were cleaned as described above (i.e., Pippin Prep and Ampure cleanups) and sequenced again on the MiniSeq.

16S sequencing data analysis was performed by the Rush Microbiome Core as previously described. In brief, microbiome bioinformatics were performed with QIIME2 2021.11 [2]. Raw sequence data were checked for quality using FastQC and merged using PEAR [3]. Merged sequences were quality filtered using the q2-demux plugin followed by denoising with DADA2 (via q2-dada2) [4]. Primer adapter sequences were removed using cutadapt algorithm. Alpha-diversity metrics (observed features, Shannon Index, Simpson's Index, and Pielou's Evenness) and beta diversity metrics were calculated using q2-diversity after samples were rarefied to a depth of 12,000 sequences per sample. Taxonomy was assigned to ASVs using the q2-feature-classifier classify-sklearn naïve Bayes taxonomy classifier against the SILVA 138 99% reference sequences database [5]. The contaminant removal software, "decontam" R package, was used to remove contaminants based on prevalence (threshold 0.5). This was performed based on prevalence of the ASVs in samples and reagent negative blank controls. Based on the above, the genera that were classified as contaminant and filtered from the dataset prior to downstream analysis (**see below**). To assess microbial community composition, we conducted a Permutational Multivariate Analysis of Variance (PERMANOVA) was conducted within QIIME2 environment with 9,999 permutations on feature levels using Aitchison distance, a linear measure of sample dissimilarity for compositional data. Differentially abundant phylum and genus level taxa between pairwise groups were identified using MaAsLin219. Functional metabolic profiles of bacterial communities were analyzed using PICRUSt2 and analysis was performed using MaAsLin2.

Genera identified as contaminant

d__Bacteria; p__Proteobacteria; c__Gammaproteobacteria; o__Vibrionales; f__Vibrionaceae; g__Photobacterium
d__Bacteria; p__Proteobacteria; c__Gammaproteobacteria; o__Pseudomonadales; f__Pseudomonadaceae; g__Pseudomonas
d__Bacteria; p__Proteobacteria; c__Alphaproteobacteria; o__Rickettsiales; f__Mitochondria; g__Mitochondria
d__Bacteria; p__Firmicutes; c__Bacilli; o__Bacillales; f__Planococcaceae; g__Chryseomicrobium
d__Bacteria; p__Deinococcota; c__Deinococci; o__Thermales; f__Thermaceae; g__Thermus; s__Thermus_thermophilus
d__Bacteria; p__Firmicutes; c__Bacilli; o__Staphylococcales; f__Staphylococcaceae; g__Staphylococcus
d__Bacteria; p__Actinobacteriota; c__Actinobacteria; o__Micrococcales; f__Microbacteriaceae; g__Microbacterium
d__Bacteria; p__Firmicutes; c__Thermoanaerobacteria; o__Thermoanaerobacterales; f__Thermoanaerobacteraceae; g__Caldanaerobacter
d__Bacteria; p__Cyanobacteria; c__Cyanobacteriia; o__Chloroplast; f__Chloroplast; g__Chloroplast
d__Bacteria; p__Actinobacteriota; c__Actinobacteria; o__Propionibacteriales; f__Nocardioidaceae; g__Marmoricola
d__Bacteria; p__Firmicutes; c__Bacilli; o__Lactobacillales; f__Aerococcaceae; g__Eremococcus; s__uncultured_bacterium
d__Bacteria; p__Proteobacteria; c__Alphaproteobacteria; o__Rhizobiales; f__Xanthobacteraceae; g__Bradyrhizobium
d__Bacteria; p__Proteobacteria; c__Alphaproteobacteria; o__Sphingomonadales; f__Sphingomonadaceae; g__Sphingomonas

References

1. Naqib A, Poggi S, Wang W, Hyde M, Kunstman K, Green SJ. Making and Sequencing Heavily Multiplexed, High-Throughput 16S Ribosomal RNA Gene Amplicon Libraries Using a Flexible, Two-Stage PCR Protocol. Methods Mol Biol. 2018;1783:149-69. doi: 10.1007/978-1-4939-7834-2_7. PubMed PMID: 29767361.
2. Bolyen E, Rideout JR, Dillon MR, Bokulich NA, Abnet CC, Al-Ghalith GA, Alexander H, Alm EJ, Arumugam M, Asnicar F, Bai Y, Bisanz JE, Bittinger K, Brejnrod A, Brislawn CJ, Brown CT, Callahan BJ, Caraballo-Rodriguez AM, Chase J, Cope EK, Da Silva R, Diener C, Dorrestein PC, Douglas GM, Durall

DM, Duvallet C, Edwardson CF, Ernst M, Estaki M, Fouquier J, Gauglitz JM, Gibbons SM, Gibson DL, Gonzalez A, Gorlick K, Guo J, Hillmann B, Holmes S, Holste H, Huttenhower C, Huttley GA, Janssen S, Jarmusch AK, Jiang L, Kaehler BD, Kang KB, Keefe CR, Keim P, Kelley ST, Knights D, Koester I, Kosciolek T, Kreps J, Langille MGI, Lee J, Ley R, Liu YX, Loftfield E, Lozupone C, Maher M, Marotz C, Martin BD, McDonald D, McIver LJ, Melnik AV, Metcalf JL, Morgan SC, Morton JT, Naimey AT, Navas-Molina JA, Nothias LF, Orchanian SB, Pearson T, Peoples SL, Petras D, Preuss ML, Pruesse E, Rasmussen LB, Rivers A, Robeson MS, 2nd, Rosenthal P, Segata N, Shaffer M, Shiffer A, Sinha R, Song SJ, Spear JR, Swafford AD, Thompson LR, Torres PJ, Trinh P, Tripathi A, Turnbaugh PJ, Ul-Hasan S, van der Hooft JJJ, Vargas F, Vazquez-Baeza Y, Vogtmann E, von Hippel M, Walters W, Wan Y, Wang M, Warren J, Weber KC, Williamson CHD, Willis AD, Xu ZZ, Zaneveld JR, Zhang Y, Zhu Q, Knight R, Caporaso JG. Reproducible, interactive, scalable and extensible microbiome data science using QIIME 2. Nat Biotechnol. 2019;37(8):852-7. doi: 10.1038/s41587-019-0209-9. PubMed PMID: 31341288; PMCID: PMC7015180.

3. Zhang J, Kobert K, Flouri T, Stamatakis A. PEAR: a fast and accurate Illumina Paired-End reAd mergeR. Bioinformatics. 2014;30(5):614-20. Epub 20131018. doi: 10.1093/bioinformatics/btt593. PubMed PMID: 24142950; PMCID: PMC3933873.

4. Callahan BJ, McMurdie PJ, Rosen MJ, Han AW, Johnson AJ, Holmes SP. DADA2: High-resolution sample inference from Illumina amplicon data. Nat Methods. 2016;13(7):581-3. Epub 20160523. doi: 10.1038/nmeth.3869. PubMed PMID: 27214047; PMCID: PMC4927377.

5. Quast C, Pruesse E, Yilmaz P, Gerken J, Schweer T, Yarza P, Peplies J, Glockner FO. The SILVA ribosomal RNA gene database project: improved data processing and web-based tools. Nucleic Acids Res. 2013;41(Database issue):D590-6. Epub 20121128. doi: 10.1093/nar/gks1219. PubMed PMID: 23193283; PMCID: PMC3531112.
